# A Framework for the Joint Placement of Edge Service Infrastructure and User Plane Functions for 5G

**DOI:** 10.3390/s19183975

**Published:** 2019-09-14

**Authors:** Irian Leyva-Pupo, Alejandro Santoyo-González, Cristina Cervelló-Pastor

**Affiliations:** Department of Network Engineering, Universitat Politècnica de Catalunya (UPC), 08860 Castelldefels, Spain

**Keywords:** 5G, edge computing, user plane function, framework, ILP, optimization problem

## Abstract

Achieving less than 1 ms end-to-end communication latency, required for certain 5G services and use cases, is imposing severe technical challenges for the deployment of next-generation networks. To achieve such an ambitious goal, the service infrastructure and User Plane Function (UPF) placement at the network edge, is mandatory. However, this solution implies a substantial increase in deployment and operational costs. To cost-effectively solve this joint placement problem, this paper introduces a framework to jointly address the placement of edge nodes (ENs) and UPFs. Our framework proposal relies on Integer Linear Programming (ILP) and heuristic solutions. The main objective is to determine the ENs and UPFs’ optimal number and locations to minimize overall costs while satisfying the service requirements. To this aim, several parameters and factors are considered, such as capacity, latency, costs and site restrictions. The proposed solutions are evaluated based on different metrics and the obtained results showcase over 20% cost savings for the service infrastructure deployment. Moreover, the gap between the UPF placement heuristic and the optimal solution is equal to only one UPF in the worst cases, and a computation time reduction of over 35% is achieved in all the use cases studied.

## 1. Introduction

5G-identified use cases and stringent technical requirements [1] are forcing a deep evolution of current communication technologies and paradigms. Network Function Virtualization (NFV), Edge Computing (EC) and Software Defined Networks (SDNs), just to mention a few examples, are driving radical technological changes that span across all 5G layers.

Among these revolutionary proposals, EC [2,3] has become the solution to upcoming ultra-low latency communication requirements (e.g., real-time services, virtual and augmented reality, etc.). Future ultra-high bandwidth use cases and envisioned service trends under 5G networks will lead to unprecedented demands for hyper/connectivity and ultra-reliable high performance. As a result, a decentralized architecture of geo-distributed computing nodes has emerged as the only feasible deployment scheme to ensure end-user demand satisfaction. However, due to the distributed nature and the expected large number of edge nodes (ENs) in a 5G and EC ecosystem (where storage, computing and networking resources are brought to the network edge), capital and operational expenditures (CAPEX and OPEX) have become critical challenges.

5G networks must be able to provide diverse services with different requirements. For instance, services under the ultra-reliable and low-latency communication (uRLLC) category require high reliability and low response time whereas others belonging to the enhanced mobile broadband (eMBB) class need high bandwidth and processing capacity. Thus, flexible and customizable deployment of the required User Plane Functions (UPFs) is necessary. Concretely, latency and reliability stringent requirements can be satisfied by placing UPFs closer to the end-users and assigning more than one UPF to the access nodes. However, this kind of approach implies an increase in the number of UPFs by a factor of 20 to 30 times the original amount [4]. A higher number of UPFs will not only increase the costs but also the occurrence of UPF relocations, mainly conditioned by users mobility and handover procedures in mobile networks. Relocations in the UPFs occur when a user equipment (UE) with an active Protocol Data Unit (PDU) session, registers in a base station (BS) that is served by a UPF different from the one linked to its source BS. Frequent relocations can affect the overall Quality of Experience (QoE) by introducing additional delays during handovers and signaling overhead for bearer establishment [5]. Therefore, the design of effective UPF placement strategies, capable of meeting the service requirements and minimizing the costs without degrading QoE is of utmost importance.

In this context, a core approach to reduce CAPEX/OPEX is the joint optimization of the EN and UPF placement strategies. A solution to this problem can exponentially increase overall deployment and operation cost savings, while effectively guaranteeing 5G service requirement satisfaction. Solving this problem is challenging due to the numerous trade-offs involved. For instance: (a) only minimizing the number of ENs and UPFs, results in performance degradation and unsatisfied end-user demands, (b) the inter-dependence between EN sizing and location-dependent costs leads to unmet requirement scenarios due to service demand variation and unexpected events (e.g., emergencies, disaster situations), (c) through the use of current in-use sites (e.g., Internet Exchange Points, IXPs) significant cost reductions can be achieved, however, if the forecasted demand does not follow the expected path, it may be better to choose other locations closer to the user demand in spite of its lack of IT capabilities, as it would considerably decrease the expenses over time.

Given this situation, this paper proposes a framework for a cost-effective deployment of both the edge service infrastructure and the required UPFs to guarantee overall cost minimization and service demand satisfaction. To that aim, our main contributions are:

- A framework proposal for the joint EN and UPF placement optimization problems.
- A novel solution approach to the UPF placement problem (UPFPP) considering optimally located edge service infrastructure, user mobility, latency and reliability requirements.

## 2. Related Work and Motivation

Two main research areas are involved in the solution of the problem presented in the previous section: Edge Node Placement and UPF Placement under 5G networking. The following sections present a comprehensive study of current literature on both topics.

### 2.1. Edge Node Placement

Extensive research has been carried out in subjects closely related to the EN placement problem (ENPP) within mobile network planning, facility location and EC implementations studies [6,7,8,9].

Wang et al. [7], propose to deploy a set of macro-cells whose coverage area is estimated based on a simplification of the underlying demand. In [10], the main target is to find the optimal locations to deploy temporal BSs during emergency situations. In both [7,10] the customer demand assumed as input is over simplified. As a result, both studies lack the flexibility to deal with highly dynamic scenarios as those found under 5G, where the demand continuously changes hindering the placement optimization process. Meanwhile, in [9] the optimum number of fog nodes (FNs) is found using stochastic geometry analysis, aiming at reducing the total number of FNs. Wang et al. article [11] consider workload efficient distribution and strong latency constraints as core parameters to choose adequate edge server locations.

The authors of [12] present an edge server placement platform that ensures the discovery of unforeseen suitable sites, by analyzing user communication patterns. Some limitations of this study are: (a) user clustering should be done base on the different use cases and typical requirements of 5G networking, including both fixed and mobile baseline data, along with an analysis of demand geo-distribution characterization, (b) 5G ultra-dense deployments and its envisioned complex service demand interrelation (due to the broad mixture of use case scenarios) has to be considered and, (c) the infrastructure sizing should comprise at least the following elements: number of users, application-based requirements, high reliability and availability margins, ultra-high bandwidth requirements. In [13], the data center placement problem is formulated seeking to minimize the costs of the entire data center network. Powerful insight for the ENPP solution is provided in this paper. However, its applicability to solve the problem presented in Section 1 is limited due to the exponential number of edge nodes to deploy in a small to medium-sized 5G service area, the communication restrictions between users and services as a result of the latency constraints, forcing the formulation of a “coverage” problem and, the absence of a proper demand characterization in order to achieve real-life optimization.

Facility Location Problems (FLPs) and server placement problems [14,15] are closely related to the ENPP. Nevertheless, traditional formulations and solutions in these fields cannot be directly applied to the ENPP since, for instance, traditional FLP formulations usually follow a particular problem type guidelines (i.e., coverage FLP) and the cost function formulation is rather simple. Furthermore, within the ENPP site-dependent characteristic analysis is mandatory given the expected density in urban deployments.

### 2.2. UPF Placement

The 5G UPFs are analogous to Service Gateways (SGWs) and Packet Gateways (PGWs) in Long-Term Evolution (LTE) networks. However, unlike SGWs and PGWs, UPFs only perform user-plane related functions. The placement of SGWs and PGWs has been addressed in a wide variety of research works [5,16,17,18,19] comprising different points of view.

The authors in [5] put forward the necessity of avoiding mobile gateway relocations through the optimal placement of SGWs over a distributed network of data centers. They formulate the optimization problem as an ILP and propose a heuristic solution based on a greedy algorithm. Their main target is to minimize the overall cost of SGW relocations subject to SGW capacity restrictions. In [16], an algorithm for creating and placing virtual instances of PGWs is proposed. Its main goal is to reduce costs while ensuring QoE to users. In this regard, the load assigned to PGWs as well as their imbalance are optimized by considering the application/service type and geographical location when selecting a PGW to attend a user’s request. The PGWs placement is modeled as a nonlinear optimization problem and three heuristics are proposed. Taleb et al. [17] introduce a set of solutions to tackle the placement of virtual gateways. Their main objective is to determine Virtual Network Functions (VNFs) best locations, so that, the path between users and PGWs is minimized while, at the same time, SGW relocations are reduced. Three placement strategies are formulated by considering delay and relocations requirements. Nonetheless, the VNF maximum capacity restriction is overlooked.

Other papers such as [18,19] address the SGWs and PGWs placement in 5G networks upon the concept of Control and User Plane Separation (CUPS). Ksentini et al. in [18] separate the SGW functionalities into SGW-U and SGW-C and define an algorithm for the SGW-C placement. This algorithm is aimed at reducing SGW relocations and at balancing their traffic load. Nevertheless, they do not take into account latency requirements for the gateway placement. In [19], the possibility of using a 5G hybrid architecture where the PGW and SGW functions could be deployed by using either VNFs or SDN elements is presented. Taking as reference this architecture, three optimization models are proposed. Their main aim is to determine the optimal sizing and planning for the data center, PGW and SGW elements. Although data and control plane latency requirements are considered, the effects of users mobility on network functions (NFs) placement are neglected.

Despite the plethora of papers tracking the placement of mobile gateways (i.e., SGWs and PGWs), the existence of a model capable of integrating aspects such as mobility, latency, reliability, costs reduction and gateway available capacity is missing. Moreover, all the works mentioned above take as reference the mobile network architectures based on LTE systems. In a previous work [20], we propose a framework to place 5G NFs and to optimize VNF infrastructure (VNFI) resources following the 3GPP 5G architecture [21]. Our main focus in this precedent research was the placement of control plane NFs, where despite the assignment of UPFs to the Session Management Functions (SMFs) was addressed, it started from the criterion that UPFs were already optimally located.

## 3. 5G Reference Architecture

The 3GPP architecture for 5G networks [21] is an evolution of the current 4G system, based on the concepts of CUPS, service-based architecture and network slicing. This architecture mainly consists of the following elements: Network Slice Selection Function (NSSF), Authentication Server Function (AUSF), Unified Data Management (UDM), Access and Mobility Management Function (AMF), Session Management Function (SMF), Policy Control Function (PCF), Application Function (AF), UPF, UE, (Radio) Access Network ((R)AN) and Data Network (DN). The separation of the control and user planes guarantees that the resources of each plane can be scaled independently and that the UPFs can be distributed and deployed near the users. This approach allows satisfying increasing traffic demands at a lower cost-per-bit and to serve low-latency applications hosted at the network edge [22].

**Figure 1:**
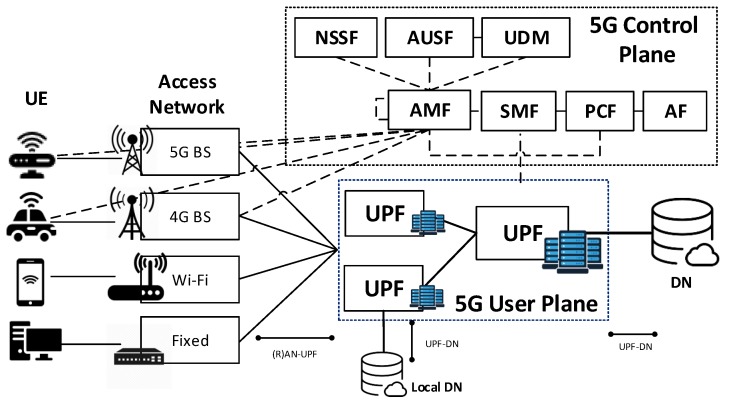
5G architecture based on CUPS.

The main component of the 5G user plane is the UPF while the control plane is formed by the NSSF, AUSF, UDM, AMF, SMF, PCF and AF. Figure 1 illustrates the 5G architecture and the interactions among its different entities. The UPFs process data plane packets between the (R)AN and the DN and act as PDU session anchor points to provide intra/inter-Radio Access Technologies (RATs) mobility. Additionally, UPFs provide packet routing and forwarding functionalities, access control, QoS handling, and lawful interception. To perform all these functions they communicate with the SMFs located in the control plane. The SMFs are responsible for selecting, controlling and managing the UPFs for the establishment of PDU sessions.

## 4. Framework Proposal

**Figure 2:**
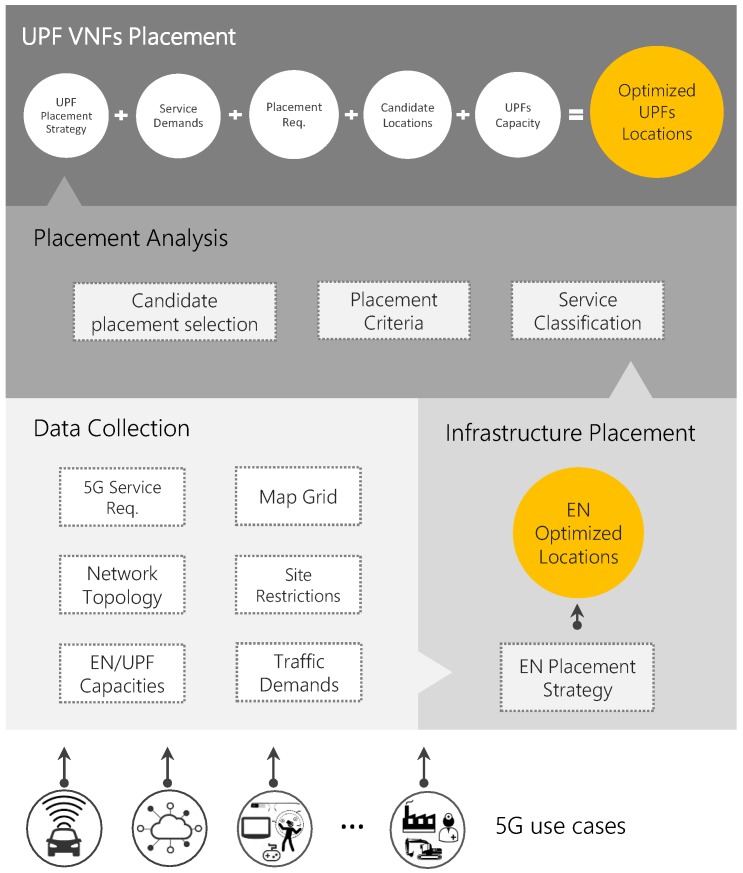
Placement framework.

To solve the problem presented in Section 1 we assume that UPFs are to be deployed in optimally placed service infrastructure (i.e., ENs). Consequently, by starting with an EN placement stage and executing the UPF placement afterwards, a feasible deployment scheme is ensured, considering that there is no interrelation between the demands and parameters on each optimization procedure. The proposed framework to jointly optimize the EN and UPFs placement is depicted in Figure 2.

**Figure 3:**
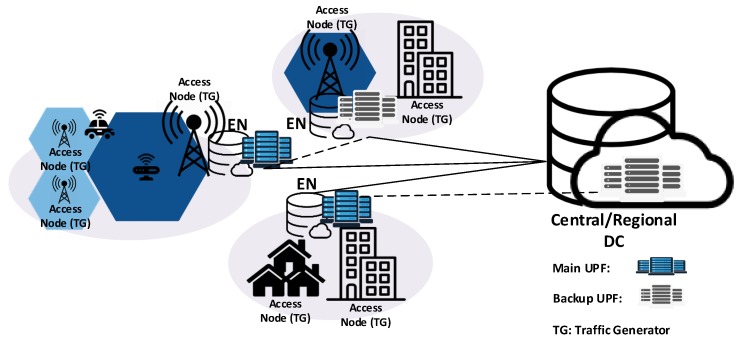
A generic representation of the framework output for the ENs and UPFs placement.

The first stage collects and processes all the input data required for the upper layers (see Table 1). Namely, 5G service requirements are passed to the platform, along with the network topology, territory of interest, EN and UPF capacities, traffic demand model and site restrictions (referred to the potential EN sites built from the network topology nodes and other potential locations, e.g., Central Offices, IXPs). The next stage deals with the placement of the edge service infrastructure while the last two phases solve the UPFPP. In Figure 3, a generic framework output is represented to clarify how the different elements of the system, i.e., access nodes (traffic generators (TGs)), EN and UPFs, may be allocated. Details of all processing stages are provided in the following subsections.

### 4.1. Edge Node Placement Stage

Overall, in this stage our goal is to place computing, storage and network resources at the edge of the network in a cost-effective manner. To decrease the EN network costs, we aim to optimize the site selection and reduce the number of ENs deployed and the capacity assigned to each EN. The underlying problem formulation models the service demand, distributed over a given area, as traffic generators (TGs) [23]. In more detail, the aggregated cell structure composed by the TGs in the form of 4G/5G mobile base stations, wireless access points, etc., is considered to be known. Moreover, since the traffic to the services hosted in any given EN is aggregated through the 5G access layer, the (R)AN nodes distributed over a given service area and the underlying demand are simplified and modeled as TGs [7,23]. This kind of model allows us to simplify the problem without loss of generality since the placement optimization is directly linked to the capacities and behavior of the traffic aggregation points (i.e., TGs) and thus independent from the particularities of the underlying end-users. The cost function for the optimization problem is presented in (1).

(1) $$\begin{array}{ccc} \mathrm{Min}\; & \sum_{\forall e}\omega \cdot c_{e}\cdot v_{e}+\sum_{\forall e,\;t}L_{et}\cdot u_{et}+\sum_{\forall e}F_{e}\cdot v_{e} & \end{array}$$

where:

- ω: cost per capacity unit
- $c_{e}$: capacity of an EN at *e*
- $v_{e}$: 1 if an EN at *e* is placed, 0 otherwise
- $L_{et}$: cost of interconnecting an EN at *e* and a TG at *t*
- $u_{et}$: 1 if a TG at *t* is covered by an EN at *e*, 0 otherwise
- $F_{e}$: fixed cost of deploying an EN at *e*

Through expression (1) the main expenses when deploying an EN network are represented: EN capacity costs (ω), TG-EN interconnection expenses and any site-specific costs. Several restrictions were added to characterize the TG service levels and underlying demands. Every TG was ensured to be fully covered (in terms of its particular demand) by one or more ENs, always considering the reliability requirement of each TG. This way, if an arbitrary TG required ultra-high reliability, the model ensured that it was covered by at least two ENs. Additionally, each EN was assigned limited capacity (a maximum capacity value was set for every EN candidate). Latency requirements were constrained through predefined thresholds looking to satisfy most of the envisioned 5G delay-sensitive use cases [1]. For this purpose, two main latency values were selected (see Section 5.1) accounting for TGs with ultra-low latency requirements (maximum delay of 1 ms) and low latency requirements.

The **Infrastructure Placement** module is in charge of the ENPP solution. At first, it carries out a partial demand distribution characterization, by classifying the service areas as urban or rural and it executes additional pre-processing optimization procedures. By conducting this kind of classification the problem complexity can be reduced, since a straightforward co-location strategy can be followed in less dense areas, placing the ENs within the TG infrastructure site. This kind of approach reduces CAPEX and OPEX as the demand in these areas is primarily scattered over large underpopulated territories. Within this stage, further pre-optimization is carried through pre-processing optimization procedures, i.e., isolation analysis, where those TGs outside the coverage range of any other EN potential location are immediately upgraded to ENs. Finally, the EN placement method is executed to find the optimized locations to place the ENs. After the ENPP is solved, the framework outputs the set of restriction-free locations where the edge infrastructure should be placed, the allocated capacities and demand covered per EN, and additional relevant data regarding the performance of the executed solution methods.

Solving the ENPP implies the analysis of all possible EN-TG combinations in order to find the minimum cost solution. Given the latency and reliability constraints and the need to satisfy all TG demands in a capacity-dependent cost model, the combinations cannot be split to reduce computation time. Additionally, ultra-dense networking and 5G strict requirements will presumably push the EN deployment to thousands of nodes in large cities. Taking this into consideration, exact methods were dropped as solutions for high-scale scenarios and two heuristic methods were implemented for performance analysis purposes: an extended and adapted variant of the **Hybrid Simulated Annealing (HSA)** presented in [23] and an **Evolutionary Algorithm** [24]. These heuristics were selected due to their flexibility when being adapted to solve heavily constrained NP-hard problems. Moreover, both evolutionary techniques and Simulated Annealing have been widely employed to solve FLPs and server placement problems [25,26,27]. The HSA in [23] was modified in order to answer the requirements of the formulation summarized in (1) and the related constraints. The results of our simulated testbed to analyze the performance of both methods can be found in Section 5.1.

### 4.2. UPF Placement Stages

By placing the UPFs at the network edge not only the network response time but also the bandwidth consumption can be significantly reduced. However, placing a UPF in every available EN results in a rise of costs and UPF relocations. Therefore, the main objective of the UPF placement stages is to determine the optimal number of UPFs and their location in the given EN infrastructure, so that 5G services requirements can be satisfied while costs are minimized. Please note that, unlike the ENPP, where the access nodes can be simplified as TGs, the UPFs placement is strongly dependant on the behaviour of their underlying end-users (e.g., service demand and mobility patterns).

The UPF placement consists of two main stages: **Placement Analysis** and **UPF VNF Placement**. The former is in charge of processing and preparing all the data required for the UPFs placement. It is a compound of three modules: *Service Classification*, *Placement Criteria* and *Candidate Placement Selection*. The *Service Classification* module aims at clustering the services into categories with similar placement requirements such as mobility, latency and reliability. For each one of these parameters, different thresholds or levels can be established. The demands of those services that belong to the same category are considered as a whole and the placement priority for each category is established.

Taking into account the service classification, *placement criteria* such as optimization objective and placement considerations are determined within the namesake module. For instance, the required number of backup UPFs for each service category is calculated according to its reliability level. The last module is responsible for selecting the candidate locations (i.e., ENs) for the UPF placement by taking into account available resources within the underlying infrastructure as well as the UPF maximum capacity and latency requirements of the service category. As a result, we obtain a set of UPF candidate locations for each access node ($N_{nc_{r}}$) and a set of near access node for each candidate ($N_{nr_{c}}$). To determine $N_{nc_{r}}$ and $N_{nr_{c}}$, clustering techniques, i.e., Fuzzy C-means (FCM), are used. This method has been widely used in the area of allocation by various researchers [28,29]. Namely, FCM is used to determine the membership preferences between access nodes-candidate and candidate-access nodes.

Finally, the **UPF VNF Placement** stage solves the UPFPP by considering service demands, placement requirements, candidate locations and UPF capacity. To address the UPFPP, we propose two strategies: an ILP model called Optimal UPF Placement (OUP) and a Near-Optimal UPF Placement (NOUP) algorithm. Table 2 summarizes the notation used in the UPF problem formulation.

#### 4.2.1. Optimal UPF Placement

The OUP model aims at reducing deployment costs by minimizing the number of UPFs. Additionally, it also deals with the operational costs related to UPF relocations. However, not all users have the same mobility patterns, being many of them static sensors or indoor that produce zero relocations. Because of this, the proposed model allows distinguishing the optimization objective by taking into account whether the UPFs to be placed will serve users with mobility characteristics (m = 1) or not (m = 0). The OUP model can be formulated as follows:

Minimize:

$$C_{cost}=\{\begin{array}{cc} \sum_{\forall c\in N_{c}}\;F_{c}\cdot \left(x_{c}+y_{c}\right)+\;\sum_{\forall c\in N_{c}}\;\sum_{\forall i\in N_{r}}\;\sum_{\forall j\in N_{r}}\;F_{h}\cdot h_{ij}\cdot a_{ijc},\;\mathrm{if}\;\mathrm{user}\;\mathrm{mobility}\;\mathrm{is}\;\mathrm{considered}\; & \;(2a)\\ \;\sum_{\forall c\in N_{c}}\;F_{c}\cdot \left(x_{c}+y_{c}\right),\;\mathrm{otherwise} & \;(2b) \end{array}$$

$$\begin{array}{cc} \; & s.t.: \end{array}$$

(3) $$\begin{array}{cccc} \; & x_{c}+y_{c}\leq 1 & \;\forall c\in N_{c} & \end{array}$$

(4) $$\begin{array}{cccc} \; & p_{rc}\leq x_{c} & \;\forall r\in N_{r},\forall c\in N_{c} & \end{array}$$

(5) $$\begin{array}{cccc} \; & b_{rc}\leq y_{c} & \;\forall r\in N_{r},\forall c\in N_{c} & \end{array}$$

(6) $$\begin{array}{cccc} \; & \sum_{\forall c\in N_{c}}p_{rc}=1 & \;\forall r\in N_{r} & \end{array}$$

(7) $$\begin{array}{cccc} \; & \sum_{\forall c\in N_{c}}b_{rc}\geq K_{u}-1 & \;\forall r\in N_{r} & \end{array}$$

(8) $$\begin{array}{cccc} \; & \sum_{\forall r\in N_{r}}d_{r}\cdot p_{rc}\leq \alpha \cdot C_{u_{max}} & \;\forall c\in N_{c} & \end{array}$$

(9) $$\begin{array}{cccc} \; & \sum_{\forall r\in N_{r}}d_{r}\cdot b_{rc}\leq C_{u_{max}} & \;\forall c\in N_{c} & \end{array}$$

(10) $$\begin{array}{cccc} \; & L_{rc}\cdot \left(p_{rc}+b_{rc}\right)\leq L_{req} & \;\forall r\in N_{r},\forall c\in N_{c} & \end{array}$$

(11) $$\begin{array}{cccc} \; & Loc_{r}=Loc_{c}\Rightarrow p_{rc}\geq x_{c} & \;\forall r\in N_{r},\forall c\in N_{c} & \end{array}$$

(12) $$\begin{array}{cccc} \; & Loc_{r}\neq Loc_{c}\Rightarrow p_{rc}\leq x_{c} & \;\forall r\in N_{r},\forall c\in N_{c} & \end{array}$$

(13) $$\begin{array}{ccc} \; & a_{ijc}=p_{ic}⊕p_{jc} & \;\forall i,j\in N_{r},\forall c\in N_{c} \end{array}$$

(14) $$\begin{array}{cccc} \; & x_{c},y_{c},p_{rc},b_{rc},a_{ijc}\;\mathrm{binary} & \;\forall r\in N_{r},\forall i,j\in N_{r},\forall c\in N_{c} & \end{array}$$

Expressions (2a) and (2b) represent UPF placement costs for services with and without mobility requirements, respectively. In (2a), the first term is associated with the cost of deploying a UPF at location *c* ($F_{c}$) while the second term is related to the UPF relocation cost ($F_{h}$). Hence, this objective function aims at optimizing both the deployment and operational expenditures by taking into account not only the number of UPFs to be deployed but the frequency of handovers in the (R)AN as well. For UPFs mostly serving users with zero or low mobility, Expression (2b) is more appropriate. In this case, the UPF placement costs can be determined in terms of the number of UPFs and their location dependent costs. Thus, the distinction of the objective function in terms of mobility requirements simplifies the problem formulation considerably when their effects on UPF relocations can be diminished.

The constraints of the system are described from (3) to (14). Expression (3) ensures that only a main or backup UPF is placed at a specific candidate location, thus guaranteeing only one UPF type for a given service category. Thus, in case of a failure in the infrastructure hosting a main UPF, its backup UPF can be instantiated in a safe location. Moreover, constraints (4) and (5) indicate that an access node must be assigned only to a candidate location where there is a main or backup UPF in place.

The constraint (6) guarantees that the demands of an access node are served by only a main UPF at a given time. In addition, restriction (7) forces the assignment of the access nodes to at least the minimum number of backup UPFs ($K_{u}$−1) necessary to satisfy the service reliability requirements. Thus, each access node will be assigned to a total of $K_{u}$ UPFs (i.e., one main UPF and $K_{u}$−1 backup UPFs). Additionally, Inequalities (8) and (9) ensure that the capacity of the main and backup UPFs is not exceeded by the underlying service demand. The factor α defines the maximum capacity to be occupied in the main UPFs by the access nodes to avoid slowing their performance.

The Expression (10) ensures that the access nodes cannot be assigned to a main or backup UPF if the latency requirement ($L_{req}$) is not satisfied. The $L_{req}$ between (R)AN nodes and UPFs is determined by taking into account their processing time ($L_{proc}$) and the service latency requirement ($L_{serv}$), $L_{req}=L_{serv}-L_{proc}$. The effects of packet transmission and queuing delays in the overall latency are considered negligible. Moreover, constraint (11) restricts the assignment of an access node to a specific main UPF if this UPF has been placed at its location. Otherwise, it can be assigned to a UPF in any other location (12). Additionally, constraint (13) is an extra restriction to take into consideration for UPF placement with mobility requirements, Expression (2a). It expresses the relationship between two access nodes and their assignment to a main UPF. Since the Expression (13) is non-linear, it must be replaced by the following inequalities: $a_{ijc}\leq p_{ic}+p_{jc}$, $a_{ijc}\geq p_{ic}-p_{jc}$, $a_{ijc}\geq p_{jc}-p_{ic}$ and $a_{ijc}\leq 2-p_{ic}-p_{jc}$. Constraint (14) indicates that $x_{c}$, $y_{c}$, $p_{rc}$, $b_{rc}$ and $a_{ijc}$ are binary variables.

#### 4.2.2. Near-Optimal UPF Placement

The OUP model for non-mobility considerations is a variant of two well known NP-hard problems, the Resilient Controller Placement Problem [30,31] and the Hierarchical Capacitated Facility Location Problems [32,33], whereas its form for mobility requirements can be seen as a combination of the previous problems and the Location Area Planning problem [5,34] which is also NP-hard. Therefore, the OUP model, in either variant, is NP-hard. Hence, the OUP approach is not feasible for ultra-dense networking scenarios where the number of access nodes and candidate locations is quite large. In these scenarios, the number of possible combinations is extremely high and finding the optimal solution for the UPFPP may require excessive computation time and resources or lead to impractical solutions. To deal with this limitation, the NOUP algorithm has been developed. The pseudocode of the NOUP solution is shown in Algorithm 1.

**Algorithm 1:** NOUP**Input**: $N_{r}$, $N_{c}$, $N_{nr_{c}}$, $N_{nc_{r}}$, $C_{u_{max}}$, Access node demands ($D_{r}$), $H_{ij}$, m, $K_{u}$ **Output**: Set of total UPFs ($S_{u_{T}}$), Set of UPFs service areas ($S_{sa_{T}}$), Set of unassigned access nodes ($S_{unassig_{T}}$) 
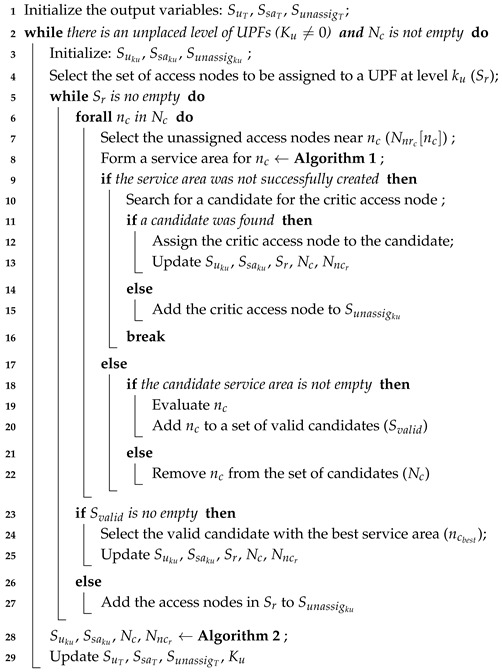


The NOUP algorithm aims at finding the best locations and number of UPFs to serve a given set of access nodes at different resilience levels. The levels of UPFs are placed according to their role, starting with the main level and ending with the last level of backup. For each level, all the available candidates are evaluated and the best ones are selected. This approach guarantees that the best locations are selected and that these locations belong to UPFs with a higher role. Executing this algorithm outputs the location of the UPFs ($S_{u_{T}}$), their service area ($S_{sa_{T}}$) and the unassigned access nodes ($S_{unassig_{T}}$), in case there are any.

The NOUP starts by initializing the output variables (line: 1). Afterwards, it proceeds to determine the UPF placement for the $K_{u}$ required levels (lines: 2–29). The first step when placing a level of UPF is to create the empty sets for the output variables in the corresponding level (i.e., $S_{u_{ku}}$, $S_{sa_{ku}}$ and $S_{unassig_{ku}}$) (line: 3). Next, the access nodes to be assigned to a level $k_{u}$ of UPFs are selected (line: 4). To determine the best location for the UPFs each available candidate is analyzed by establishing its potential service area (lines: 6–22). This kind of area is formed by the unassigned access nodes near the candidate ($N_{nr_{c}}\left[n_{c}\right]$) that satisfy the latency requirement (lines: 7–8).

At this point, Algorithm 2 is executed, which is in charge of setting the UPF service area. Its first step is to ensure that restriction (11) of the problem formulation is satisfied (lines: 1–3). To achieve this, the algorithm checks whether the candidate is a main UPF co-located with an access node (line: 1). If this is the case, the (R)AN node is assigned to the service area and the UPF available capacity ($C_{u_{c}}$), the unassigned access nodes near the candidate ($N_{nr_{c}}\left[n_{c}\right]$) and the UPF service area ($S_{sa_{c}}$) are updated (lines: 2–3). Afterwards, the unassigned (R)AN nodes near the candidate ($N_{nr_{c}}\left[n_{c}\right]$) are sorted by their proximity to the candidate (line: 4) and an assignment process takes place (lines: 5–21). This process is repeated while the UPF candidate has available capacity and there are unassigned access nodes near it.

**Algorithm 2:** FormingServiceArea

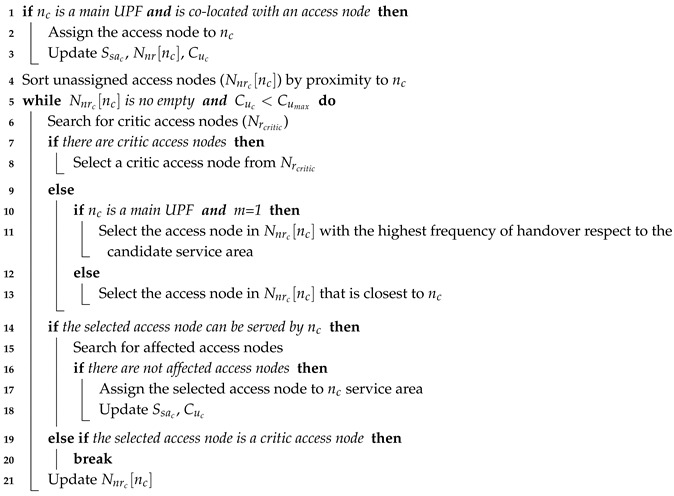



For the assignment, those access nodes that have the selected $n_{c}$ as unique candidate, known as critic access nodes ($n_{r_{critic}}$), are prioritized (lines: 7–8). If there are no critic access nodes the selection of an access node for its assignment to the candidate is made according to the UPF type and mobility requirements (lines: 9–13). Namely, if the service area belongs to a main UPF with mobility requirements (m = 1), the unassigned access node with the highest frequency of handover, with respect to the access nodes in the UPF service area, is selected (lines: 10–11). Otherwise, the chosen access node is the closest one to the candidate (lines: 10–13).

Once an access node is selected, it is necessary to check that it can be served by the UPF (line: 14). If the UPF has enough available capacity to serve the selected access node, the next step is to verify that no access node is affected by the assignment (line: 15). An assignment may affect other access nodes when their corresponding main UPF cannot be placed without violating constraint (11). If a critic access node cannot be served by the available capacity in the candidate, an error indicator is activated and Algorithm 2 is interrupted (lines 20). Otherwise, the set $N_{nr_{c}}\left[n_{c}\right]$ is updated by removing the selected access node (line: 21), regardless of whether it was assigned or not.

After Algorithm 2 is executed, the next step in Algorithm 1 is to verify whether the candidate service area was successfully created. If the execution of Algorithm 2 was interrupted due to the existence of an unassigned critic access node, a sub-process to find a candidate for the critic access node is launched (line: 10). If a candidate is found the node is assigned and the sets of UPF locations ($S_{u_{ku}}$), UPF service areas ($S_{sa_{ku}}$), unassigned (R)AN nodes ($S_{r}$), candidate locations ($N_{c}$) and candidate near the access nodes $N_{nc_{r}}$ are updated (lines: 11–13). Otherwise, the critic access node is added to the set of unassigned access nodes ($S_{unassig_{ku}}$) (lines: 14–15). In both cases, the candidate evaluation process is interrupted and restarted (line: 16). If Algorithm 2 was successfully executed, the candidate service area is inspected. If the service area is not empty, the candidate is evaluated and added to a list of valid candidates ($S_{valid}$) (lines: 18–20). The candidate evaluation is made by taking into account metrics such as utilization, worst delay and relocation avoidance for mobility considerations. If the candidate has no assigned access nodes, it is removed from the set of candidates (lines: 21–22).

Once all the candidates are analyzed, the valid candidate with the best service area is selected (lines: 23–25). The best candidate is the one that has more access nodes assigned and avoids the greatest number of UPF relocations if m = 1. Each time that the best candidate is chosen, the sets $S_{u_{ku}}$, $S_{sa_{ku}}$, $S_{r}$, $N_{c}$ and $N_{nc_{r}}$ are updated (line: 25). In case no valid candidates, the remaining access nodes in $S_{r}$ are added to the set of unassigned access nodes (lines: 26–27) and further analysis is required. These access nodes could be assigned to a UPF by relaxing the latency requirement or by deploying additional infrastructure. Notice, that these approaches imply either QoS degradation or the incurrence of additional costs. The candidate evaluation process (lines: 5–25) is repeated while there are unassigned access nodes.

Algorithm 3 is executed (line: 28) after a level $k_{u}$ of UPFs is placed. Its main objective is to reduce the number of UPFs and the relocation occurrence if mobility requirements are considered. Hence, Algorithm 3 first step is to determine the overall available capacity ($C_{u_{available}}$) in the UPFs of the level (line: 1). This capacity is compared with the UPF maximum capacity (line: 2). If the overall available capacity is higher than the UPF maximum capacity, the number of UPFs could be reduced (by removing some of them). To delete the lowest utilized UPFs, they are sorted and analyzed in descending order according to their available capacity (line: 3). For each UPF at level $k_{u}$, it is checked whether all its assigned access nodes can be served by other UPFs (lines: 5–12). If an access node can be reassigned to other UPF, this is indicated by marking the access node (lines: 7–8). If all the access nodes in a UPF service area can be served by other UPFs, they are reassigned and the UPF is deleted from the set of UPFs (lines: 9–11). As a result of this process, the set of UPFs at level $k_{u}$, their service areas, the available candidates and the candidate locations for each access node are updated (line: 12).

**Algorithm 3:** Reassignment

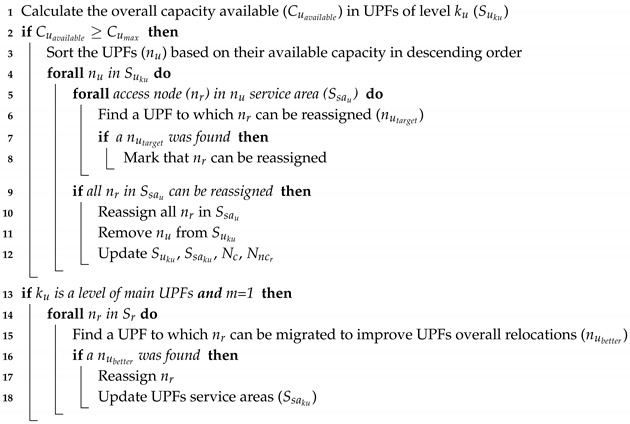



Moreover, for a level of main UPFs with mobility requirements, Algorithm 3 tries to reduce the occurrence of relocations (lines: 13–18). For each access node that has been assigned during this level, it searches for whether there is a better UPF (lines: 15). If a better UPF in terms of relocations avoidance is found, the access node is reassigned and the UPF service areas are updated (lines: 16–18). At the end of this algorithm, two additional steps can be included to ensure that each access node is assigned to its nearest UPF and to reduce the load imbalance.

Finally, in Algorithm 1, the output sets are updated and the level indicator is decremented (line: 29). Steps 2–29 are repeated while there are available candidates and levels of UPFs pending for placement.

## 5. Evaluation and Results

A territory of interest with a size of 10,000 km${}^{2}$ was used, where an arbitrary set of TGs was randomly deployed mimicking a fairly realistic demand distribution, with high density urban areas and lower traffic density in rural ecosystems. The TGs represent fixed and radio access nodes with traffic demands between 0 and 1 Tb/s. In the urban scenarios, the radio access nodes are centralized Baseband Units (BBUs) with a maximum coverage area of 3 km whereas in the rural area they are distributed BBUs with radii of 10 and 20 km. To generate the TG traffic demands, six services with different requirements of bandwidth, reliability and latency were considered [35,36]. Table 3 shows the selected use cases and their requirements.

### 5.1. ENs Placement Results

For the ENPP solution, the latency constraints were translated into Euclidean distances considering the propagation times of a direct link between any TG-EN pair. Namely, for ultra-low latency requirements, a lower bound was fixed in 2 km while the upper bound was set to 6 km (considering an approximate propagation time of 5 μs/km [19]), for ultra-low latency requirements under 1 ms and low latency requirements around 5 ms.

**Figure 4:**
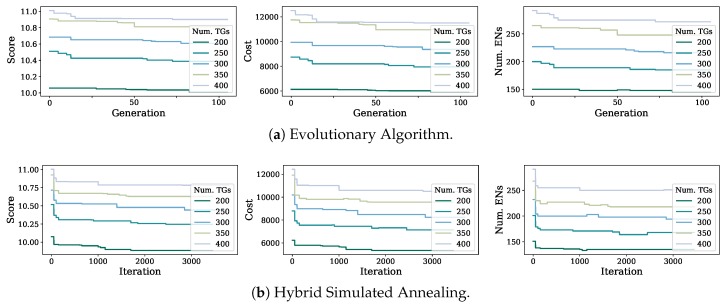
Evaluation of the proposed heuristics to solve the ENPP.

The results for the conducted experiments are shown in Figure 4a,b. Both the Evolutionary Algorithm (EA) and the HSA were tested for an arbitrary range of TGs varying between 200 and 400 (considering a representative number of nodes for envisioned 5G networks in mid to large city deployments). The hardware used to run the experiments has a 3.30 GHz CPU, x64 architecture (with 10 physical cores and 2 threads per core) and 64 GB RAM. The set of input parameters used for each algorithm is presented in Table 4 and Table 5.

Figure 4a,b both show the score, cost and number of ENs deployed by each placement strategy for all input TG values. To estimate the score, the procedure in [23] was followed. In summary, the Score plots (leftmost plots in Figure 4) demonstrate that HSA outperformed EA significantly (considering that a logarithmic scale was used to normalize the score estimation values). Namely, HSA achieved cost savings over 15% when compared to EA in every case, reaching around 20%–30% for more than 300 TGs. Similar values can be observed for the number of ENs deployed by each mechanism (rightmost plots in Figure 4), where the HSA reached a maximum of over 30% less ENs deployed.

We strongly believe these results prove the complexity of the ENPP and the limitations to be considered in order to select an adequate solving heuristic. In this particular case, when applying EA to the ENPP, the coverage nature of the problem forces a high probability of occurrence for a “dominoes effect”, where a continuous EN-TG re-arranging is caused after changing a previously selected EN-TG pairing solution. Since the node density is significantly high (as expected in future 5G networks), changing a valid EN-TG assignment, i.e., through the mutation and crossover techniques applied by evolutionary techniques, commonly results in a large chain of EN-TG reassignments throughout the complete service area. As a consequence, invalid solutions are typically generated and “repair” procedures have to be executed. This situation leads to a lower probability of a child solution enhancing a parent valid EN placement. The cost savings achieved by the placement strategies, in particular by the HSA, are directly linked to the EN network deployment costs. Nevertheless, our problem formulation and solving scheme ensure a significant reduction in the operating expenses as well due to the capacity assignment optimization and the reduction in the total number of ENs deployed.

### 5.2. UPF Placement Results

For the UPF placement solutions, a scenario composed of 100 access nodes (TGs) and the set of EN locations obtained from the HSA were selected. To evaluate the performance of our solutions regarding user mobility considerations, similar placement conditions are mandatory. Hence, the use cases presented in Table 3 were classified by taking into account only latency and reliability requirements. Two service categories were obtained as a result of the placement analysis stage. The first group comprises services with the highest requirements, i.e., automated factories, mIoT and cooperative sensing, whereas the second one is formed by the remaining services. Notice that in both groups there are services with different mobility requirements.

For the UPFs dedicated to services with high requirements, we assumed 1 ms for the overall user plane latency and 1 backup UPF ($K_{u}$ = 2). In addition, for the second group, we considered 5 ms and no backup deployment ($K_{u}$ = 1). The required number of UPF ($K_{u}$) to which an access node must be assigned was calculated upon expression (14), where $p_{r}$ and $p_{u}$ represent the failure probability of access nodes and UPFs, respectively. To achieve a service reliability greater than 99.999%, $p_{r}$ must be lower than $10^{-5}$ whereas $p_{u}$ can take any value. Specifically, $p_{r}=10^{-6}$ and $p_{u}=10^{-3}$ were assumed. Additionally, to meet latency demands of 1 ms, the overall delay (Round-Trip-Time, RTT) between access nodes and UEs cannot exceed 0.5 ms [37]. Taking this into account and assuming that UPFs and DN (application servers) are co-located with a total processing time of 0.3 ms, a maximum of 0.2 ms RTT ($L_{req}$) between access nodes and UPFs is assumed. For the second group of services, this value was relaxed to 1 ms.

(15) $$\begin{array}{c} R\left[r\right]=\left(1-\;p_{r}\right)\left[1-\prod_{\forall u\in K_{u}\left[r\right]}\left[1-(1-p_{u})\right]\right] \end{array}$$

Since the selected scenario is formed by three regions with different characteristics (i.e., City_1, City_2 and Rural), we ran our UPF placement solutions in all of them separately. Table 6 describes some characteristics of these scenarios, such as access node ($N_{r}$) and candidate location ($N_{c}$) distribution and total access network demands per service category. The candidate locations comprise the set of ENs and existent Points of Presence (PoPs). The PoPs were analyzed as candidates only for UPFs serving the second group of services. These candidates were preferred over ENs when the ENs were not able to comply with the restriction (11).

The UPF placement solutions were analyzed for different values of UPF capacity taking into account the resources available in the underlying ENs. First, the UPFs placement for services with high requirements was determined and after having updated the infrastructure available resources, we proceeded to the placement of the second group of UPFs. The results obtained by the optimal and heuristic solutions for mobility and non-mobility considerations (i.e., OUP_M1, OUP_M0, NOUP_M1 and NOUP_M0) were compared regarding the number of UPFs, UPF utilization, UPF relocations and execution time. The mathematical models were implemented with the Python-based package Pyomo [38] along with Gurobi [39] as underlying solver. Their optimal solutions were determined with zero optimality gap.

#### 5.2.1. Number of UPFs

**Figure 5:**
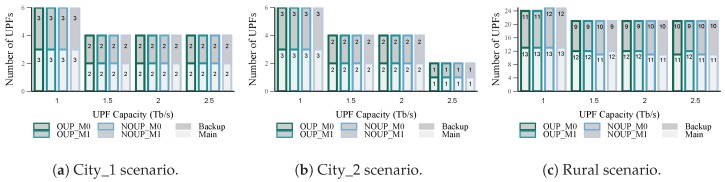
Number of UPFs vs. capacity for services with high requirements.

**Figure 6:**
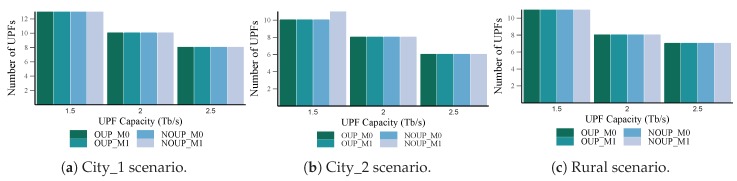
Number of UPFs vs. capacity for services with low requirements.

Figure 5 and Figure 6 depict the total number of UPFs obtained for services with high and low requirements respectively. From these figures we can observe that the proposed heuristic performance is close to the optimal solution results, for either existing mobility and not existing mobility considerations. Namely, for services with high requirements in urban scenarios (see Figure 5) the four solutions obtained the same number of UPFs (main and backup). Similar results were achieved in the rural area, but in this case, the heuristics required one UPF more than the optimal for $C_{u}$ = 1 Tb/s. Moreover, in this scenario, the number of main UPFs is always higher than the number of backups. This is because of the existence of two isolated access nodes that cannot be assigned to a backup UPF without violating the latency constraint or the main-backup relationship (see Section 4.2.2 for more details). For the second group of services (see Figure 6) all the solutions had similar results for all the scenarios and values of capacity considered, except for $C_{u}$ = 1.5 Tb/s in City_2, where NOUP_M1 obtained one UPF more than the other three methods.

In general, a reduction in the number of UPFs with the increase of their capacity can be noticed. This result is more remarkable for the second group of services where the latency requirement is less strict. Furthermore, almost all the access nodes (with the exception of two isolated rural access nodes) were assigned to the required number of UPFs according to the reliability levels established. This showcases the assertiveness of the proposed strategies for the edge infrastructure and UPFs placement.

#### 5.2.2. UPFs Utilization

**Figure 7:**
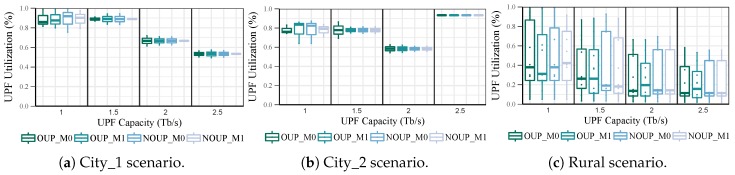
UPF Utilization vs. capacity for services with high requirements.

The load distribution was measured only in the main UPFs because the backups do not have any access node assigned in normal network conditions. For services with high requirements in urban scenarios, the four solutions provide similar levels of UPF utilization for all values of capacity, as shown in Figure 7. Moreover, the load distribution in the UPFs is quite even, being the maximum load imbalance below 25%. On the contrary, in the rural scenario (see Figure 7c) the imbalance is always above 50% and it reaches almost 100% for $C_{u}$ = 1 Tb/s. Furthermore, the UPFs average utilization is quite low with values between 10% and 40%. This behaviour is caused by isolated access nodes with low user density and service demands. Due to these results in the rural scenario, the capacity of the UPFs was readjusted by considering a granularity factor of 0.25 Tb/s.

**Figure 8:**
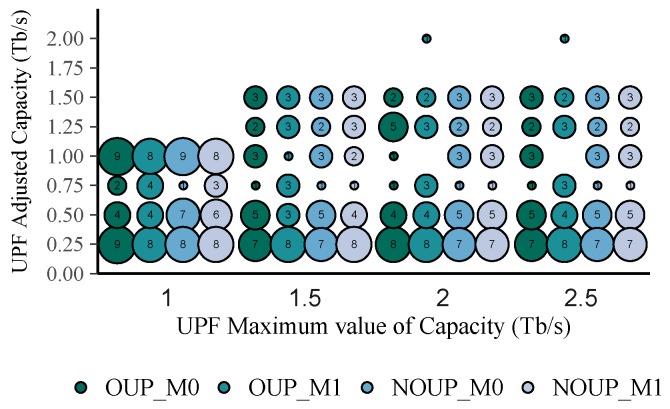
UPF adjusted capacities vs. maximum capacity for services with high requirements.

**Figure 9:**
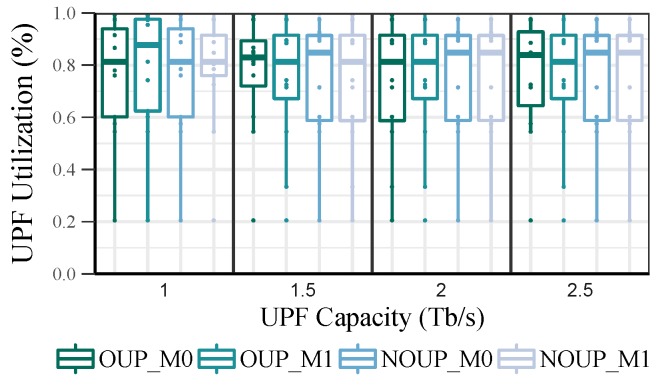
UPF Utilization distribution in rural scenario after adjusting their capacity.

Figure 8 represents the total number of UPFs obtained for each value of adjusted capacity. As it can be appreciated, most of the UPFs require low values of capacity, i.e., between 0.25 and 0.5 Tb/s. Moreover, regardless of the UPFs maximum capacity increase, their adjusted capacity remains below 1.5 Tb/s most of the time. Taking into consideration these new capacity values, the UPF utilization in the rural scenario was recalculated. After readjusting the UPFs capacity, their average utilization (see Figure 9) is always above 80% while their minimum utilization is higher than 20%. Nevertheless, the load imbalance is still quite large, with values between 45% and 80%. This metric could be further improved by considering a smaller granularity factor such as 0.1 Tb/s.

**Figure 10:**
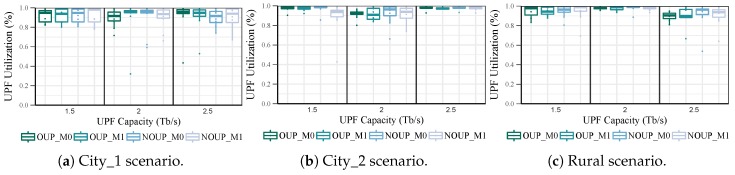
UPF Utilization vs. capacity for services with low requirements.

In Figure 10, the UPF utilization for the second group of services is shown. The four solutions provide similar results in all the scenarios with an average UPF utilization above 90%. Moreover, the load distribution in the UPFs is quite balanced with the maximum imbalance below 25%. However, most of these UPFs are overloaded with a utilization ratio above 95%. This parameter can be improved by increasing the value of the α factor, but at the cost of affecting the number of UPFs.

It can be observed from Figure 7 and Figure 10 that there is no significant difference in the UPF load distribution for the placement solutions with and without mobility consideration for either OUP or NOUP.

#### 5.2.3. UPFs Relocations

The UPF relocations rate was determined by considering the service data rate and the frequency of handovers between the radio access nodes (i.e., BBUs). For each use case, a probability of handover was established according to their user mobility requirements and type of region.

**Figure 11:**
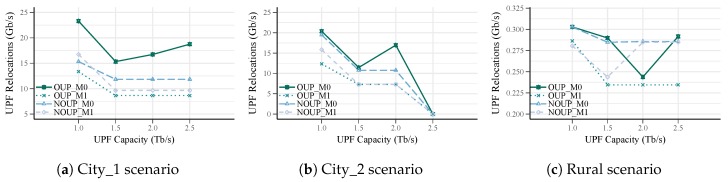
UPF Relocation Rate vs. capacity for services with high requirements.

**Figure 12:**
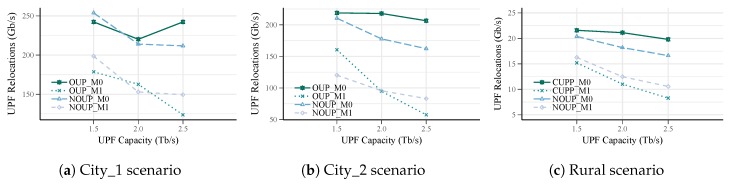
UPF Relocation Rate vs. capacity for services without low requirements.

In Figure 11 and Figure 12, the rate of UPF relocations for services with high and low requirements are depicted. At first glance, it can be observed that OUP_M1 and NOUP_M1 provide better results in comparison to OUP_M0 and NOUP_M0, even though they all have the same number of UPFs. Specifically, OUP and NOUP for mobility considerations achieve a reduction of the UPF relocations rate of up to 55% and 32%, respectively, in comparison to UPF placement without mobility (see Figure 11). For services with low requirements, this difference is higher, reaching values up to 72% and 57%, respectively.

These results are expected as in OUP_M1 and NOUP_M1 the main objective is not only to minimize the number of UPFs but also the relocation occurrence. It can be noticed that most of the time NOUP_M1 provides similar results to OUP_M1. The difference among solutions with and without mobility considerations is more remarkable for the second group of services where the rate of relocations is much higher. This is because in this category there are more services with mobility requirements and therefore the effects of this parameter are more pronounced.

Overall, a reduction of UPF relocations with the increase of the capacity can be observed. This is more noticeable for the first group of services in City_2 region where relocations are completely reduced for $C_{u}$ = 2.5 Tb/s. In this case, the occurrence of relocations is zero because all the access nodes are served by the same UPF, see Figure 11b. However, it must be highlighted that a lower number of UPFs does not necessarily imply lower relocations, but it is also mandatory to take into account users mobility patterns.

#### 5.2.4. Complexity Analysis and Execution Time

Finally, the four solutions are compared in terms of their complexity and execution time. The computational complexity of a mathematical model is given by the size of the problem which is expressed in terms of the number of variables (n) and constraints (m). However, formulating a function that relates both terms is hardly possible since this is an open issue [40]. In the worst-case, the computational complexity of the simplex algorithm was shown to be exponential in the size of the problem whereas in practice it has been found to be nxm [41]. Please note that there is a substantial difference between both approaches. Hence, the problem complexity will be formulated based on the number of variable and constraints separately and non-fixed expression will be given to establish their relationship. Table 7 characterizes the complexity of OUP for mobility and non-mobility considerations. From this table, it can be observed that the complexity of OUP_M0 and OUP_M1 in terms of their variables or constraints are O($|N_{r}\left|*\right|N_{c}|$) and O($|N_{r}{|}^{2}*\left|N_{c}\right|$), respectively. Therefore, the complexity of OUP_M0 model is asymptotically smaller than that of OUP_M1.

Regarding the computational complexity of NOUP, it is determined by the main loop of Algorithm 1. This while loop will be executed $K_{u}$ times, being $K_{u}$ the levels of UPFs that must be placed. The complexity associated with the iterative processes inside this loop is given by the number of access nodes that must be assigned ($S_{r}$) and the number of candidate locations ($N_{c}$) which are evaluated in the innermost loop of Algorithm 1. A significant time-consuming part related to the candidate evaluation process is the execution of Algorithm 2. The time complexity of Algorithm 2 is linked to its while loop which can be specified as O(|A|), where A denotes the maximum number of access nodes that can be assigned to a candidate. Please note that A cannot be found beforehand since it depends on several factors such as the number of access nodes near a candidate ($N_{nrc}\left[nc\right]$), the UPF capacity and access node demands. Hence, the overall NOUP algorithm complexity is formulated based on it. Considering the worst time-consuming scenario where only one access node is assigned to a candidate in each iteration and assuming that all the access nodes must be assigned ($S_{r}=N_{r}$) to $K_{u}$ levels of UPFs, the complexity of NOUP can be defined as O($|K_{u}\left|*\right|N_{r}\left|*\right|N_{c}\left|*|A\right|$) where $A<<N_{r}$. By comparing this complexity function with any of the OUP complexity expressions, that jointly embrace variables and constraints terms, it can be concluded that NOUP complexity is smaller.

The computation time of the proposed solutions is summarized in Table 8. The first observation made from Table 8 is that the OUP_M1 model has the worst performance with running times in the order of thousands of seconds. This higher timescale makes it unfeasible for online placement. On the contrary, the running time of the other solutions is much lower, with typical values around 1s. Concretely, the average time of NOUP in urban and rural scenarios are 0.15 s and 0.33 s, respectively, whereas OUP_M0 requires 0.95 s in the cities and 0.52 s in the rural area. Thus, our heuristic provides a running time reduction of around 85% and 35% in urban and rural areas respectively, with respect OUP_M0. These results verify that NOUP outperforms the optimal solution. Regarding the effects of mobility consideration on the heuristics execution time, there is no significant difference, being this parameter always below 0.6 s with a maximum variation of 0.25 s.

## 6. Conclusions

In this paper, we provided a framework to optimize the UPFs placement based on a previous optimal location of the edge service infrastructure (i.e., ENs). The main goal was to determine the optimal number and location of ENs and UPFs to meet 5G stringent requirements in a cost-effective manner.

A thorough evaluation of the proposed placement solutions was presented. To optimize the EN placement, two strategies were proposed considering their proven use in similar problems. Overall, HSA outperformed the evolutionary method tested achieving cost savings over 20% due to its adaptability to the problem and its better exploration of the solution space. These approaches enhance the UPF placement results by reducing their deployment costs and improving QoS since the UPF candidate locations (i.e., ENs) are optimally determined according to users’ traffic demands.

Regarding the UPFs placement, the devised solutions allow minimizing not only the deployment costs (measured in terms of the number of UPFs) but also the operational costs related to UPF relocations. Concretely, regarding user mobility, the UPF solutions regarding user mobility allowed reductions in the UPF relocation rate of up to 55% and 70% for services with high and low requirements, respectively, at the same deployment cost. Moreover, the proposed heuristic for UPF placement with mobility considerations provided significant run-time improvements, while keeping similar cost values when compared to the optimal. The obtained results demonstrate the assertiveness of the proposed approaches in attaining their objectives. Thus, this framework is a valuable tool for 5G network operators to efficiently plan and deploy their resources.

Future research should focus on the simultaneous and online solution of both optimization problems (i.e., ENPP, UPFPP), comparing sequential and simultaneous approaches on 5G emulated scenarios or testbeds.

## Figures and Tables

**Table 1:** Framework input data.

Parameter	Meaning
Network_Topology	Set of (R)AN elements and their coordinates
Service_Reqs.	5G service requirements of latency, mobility and reliability
Traffic_demands	Traffic demands in the access nodes
$EN/UPF_{capacities}$	EN and UPF available capacities specifying at least its maximum value
Map_Grid	Territory of interest where (R)AN elements are located
Site_Restrictions	Non-technical restrictions affecting EN suitable locations

**Table 2:** Notation used in the UPFPP formulation.

Notation	Description
**Sets and Parameters**	
$N_{r}$	Set of access nodes (TG)
$N_{c}$	Set of UPF candidate locations (EN)
$d_{r}$	Traffic demand at each access node
$C_{u_{max}}$	Maximum capacity of each UPF
α	Percentage of capacity to be used in the main UPFs
$F_{c}$	Fixed cost of deploying an UPF at candidate node *c*
$F_{h}$	Cost associated with UPF relocations
$h_{ij}$	Frequency of handovers between access nodes *i* and *j*
$K_{u}$	Minimum number of UPF per access node
$L_{rc}$	Latency between access nodes and candidates
$L_{req}$	Latency requirement between access nodes and UPFs
*m*	Mobility requirement indicator
**Binary variables**	
$x_{c}$	1 if there is a main UPF installed at candidate node *c*
$y_{c}$	1 if there is a backup UPF installed at candidate node *c*
$p_{rc}$	1 if access node *r* has a main UPF installed at node *c*
$b_{rc}$	1 if access node *r* has a backup UPF installed at node *c*
$a_{ijc}$	1 if access node i or j is assigned to a main UPF installed at candidate node *c*

**Table 3:** 5G service requirements.

Service	Latency	Data Rate	Density	Reliability	m
	(ms)	(Mb/s)	(users/km${}^{2}$)	(%)	
Automated Factories	≤1	1	$10^{4}$ (R), 0 (U)	99.999	0
mIoT	≤ 1	1	$10^{3}$ (R), $10^{4}$ (U)	99.999	0/1
Cooperative Sensing	≤1	5	10 (R), 100 (U)	99.999	1
Home & Office	≤10	50 (R), 300 (U)	100 (R), $10^{3}$ (U)	90	0
Traffic Efficiency	≤5	25	5 (R), 50 (U)	90	1
50 Mb/s everywhere	≤10	50	50 (R), 400 (U)	90	1

**Table 4:** Input parameters for the Evolutionary Algorithm.

Parameter	Value
Num. Generations	100.00
Num. Individuals	100.00
Mutation rate	0.0100

**Table 5:** Input parameters for the Hybrid Simulated Annealing.

Parameter	Value
Minimum Temperature	0.0001
Maximum Temperature	1.0000
Temperature Iterations	10.000
Fast Alpha	0.8000
Slow Alpha	0.9500
Num. Neighbors	10.000

**Table 6:** Network nodes distribution.

Region	Candidate Nodes		Access Nodes		Total Demand (Tb/s)	
	EN	PoP	Radio	Fixed	Group 1	Group 2
City_1	13	12	10	22	2.67	17.93
City_2	12	12	11	21	2.34	14.62
Rural	33	0	16	20	6.34	15.66

**Table 7:** OUP Complexity Analysis.

Model	Time Complexity	
	Variables	Constraints
OUP_M0	$2*|N_{r}\left|*\right|N_{c}\left|+2*\right|N_{c}|$	$5*|N_{r}\left|*\right|N_{c}\left|+3*\right|N_{c}\left|+2*\right|N_{r}|$
OUP_M1	$|N_{r}{|}^{2}*|N_{c}\left|+2*\right|N_{r}\left|*\right|N_{c}\left|+2*\right|N_{c}|$	$|N_{r}{|}^{2}*|N_{c}\left|+5*\right|N_{r}\left|*\right|N_{c}\left|+3*\right|N_{c}\left|+2*\right|N_{r}|$

**Table 8:** Execution Time.

Scenario	Model	Execution Time (s)						
		$C_{u}$ for Group 1				$C_{u}$ for Group 2		
		1.0	1.5	2.0	2.5	1.5	2.0	2.5
City_1	OUP_M0	3.41	0.37	0.43	0.45	1.11	1.18	0.47
	OUP_M1	10,428	8352	537	2378	244	190	121
	NOUP_M0	0.11	0.11	0.11	0.12	0.17	0.17	0.10
	NOUP_M1	0.16	0.14	0.15	0.13	0.21	0.16	0.13
City_2	OUP_M0	3.16	0.43	0.45	0.38	0.56	0.52	0.48
	OUP_M1	36,065	17192	4757	5.73	1420	176	30,058
	NOUP_M0	0.10	0.12	0.14	0.08	0.17	0.11	0.09
	NOUP_M1	0.12	0.14	0.14	0.14	0.16	0.14	0.12
Rural	OUP_M0	0.61	0.59	0.52	0.57	0.58	0.51	0.32
	OUP_M1	13.30	13.15	13.04	13.13	20,440	182,811	526
	NOUP_M0	0.37	0.36	0.33	0.29	0.33	0.25	0.09
	NOUP_M1	0.40	0.29	0.33	0.31	0.56	0.43	0.18
